# Poly[[tetra­aqua­(μ_4_-imidazole-4,5-dicarboxyl­ato)(μ_3_-imidazole-4,5-dicarboxyl­ato)-μ_3_-sulfato-μ_2_-sulfato-cobalt(II)dierbium(III)] monohydrate]

**DOI:** 10.1107/S1600536810047203

**Published:** 2010-11-20

**Authors:** Feng Sun

**Affiliations:** a School of Chemistry and Environment, South China Normal University, Guangzhou 510631, People’s Republic of China

## Abstract

The asymmetric unit of the title compound, {[CoEr_2_(C_5_H_2_N_2_O_4_)_2_(SO_4_)_2_(H_2_O)_4_]·H_2_O}_*n*_, contains a Co^II^ ion, two Er^III^ ions, two imidazole-4,5-dicarboxyl­ate (imdc) ligands, two SO_4_^2−^ anions, four coordinated water mol­ecules and one uncoordinated water mol­ecule. The Co^II^ ion is six-coordinated by two O atoms from two coordinated water mol­ecules and two O atoms and two N atoms from two imdc ligands in a slightly distorted octa­hedral geometry. Both Er^III^ ions are eight-coordinated in a bicapped trigonal–prismatic coordination geometry. One Er^III^ ion is coordinated by four O atoms from two imidazole-4,5-dicarboxyl­ate ligands, three O atoms from three SO_4_^2−^ anions and one water O atom; the other Er^III^ ion is bonded to five O atoms from three imdc ligands, two O atoms from two SO_4_^2−^ anions as well as one coordinated water mol­ecule. The coordinated metal units are connected by bridging imdc ligands and sulfate ions, generating a two-dimensional heterometallic layer. The two-dimensional layers are stacked along the *b* axis *via* N—H⋯O, O—H⋯O and C—H⋯O hydrogen-bonding inter­actions between water mol­ecules, SO_4_^2−^ anions, and imdc ligands, generating a three-dimensional framework.

## Related literature

For applications of lanthanide–transition metal complexes similar to the title compound, see: Cheng *et al.* (2006 ▶); Kuang *et al.* (2007 ▶). For related structures, see: Sun *et al.* (2006 ▶); Zhu *et al.* (2010 ▶). 
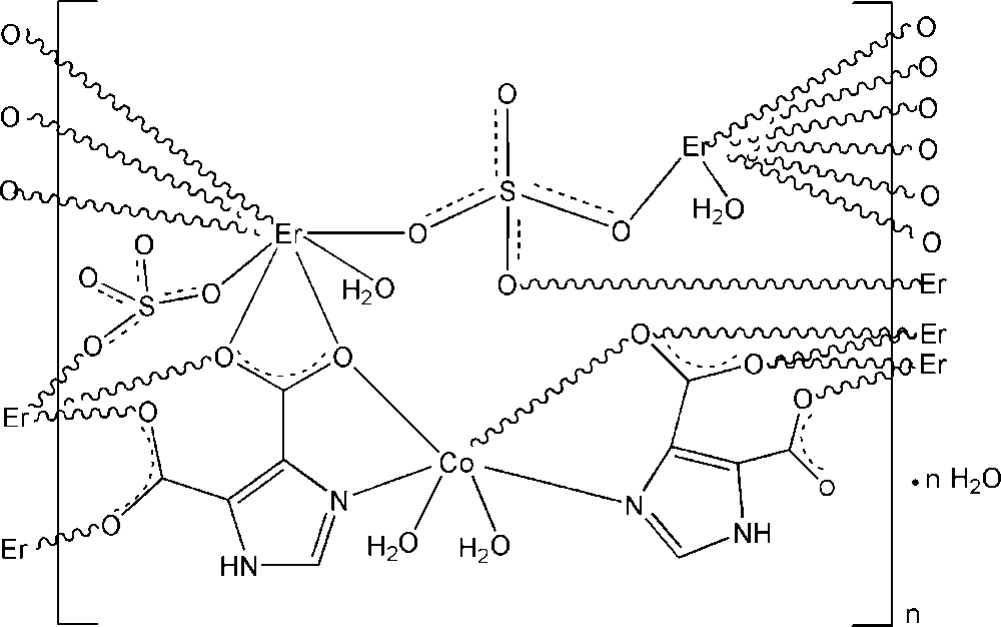


## Experimental

### 

#### Crystal data

[CoEr_2_(C_5_H_2_N_2_O_4_)_2_(SO_4_)_2_(H_2_O)_4_]·H_2_O
*M* *_r_* = 983.84
Triclinic, 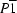

*a* = 9.0512 (5) Å
*b* = 10.6827 (6) Å
*c* = 12.8945 (8) Å
α = 92.955 (1)°
β = 97.054 (1)°
γ = 108.612 (1)°
*V* = 1167.17 (12) Å^3^
*Z* = 2
Mo *K*α radiation
μ = 8.12 mm^−1^
*T* = 296 K
0.20 × 0.18 × 0.15 mm

#### Data collection

Bruker APEXII area-detector diffractometer
Absorption correction: multi-scan (*SADABS*; Sheldrick, 1996 ▶) *T*_min_ = 0.215, *T*_max_ = 0.296
6026 measured reflections
4123 independent reflections
3820 reflections with *I* > 2σ(*I*)
*R*_int_ = 0.017

#### Refinement

*R*[*F* ^2^ > 2σ(*F* ^2^)] = 0.023
*wR*(*F* ^2^) = 0.055
*S* = 1.03
4123 reflections
397 parameters
12 restraints
H atoms treated by a mixture of independent and constrained refinement
Δρ_max_ = 0.87 e Å^−3^
Δρ_min_ = −1.35 e Å^−3^

### 

Data collection: *APEX2* (Bruker, 2004 ▶); cell refinement: *SAINT* (Bruker, 2004 ▶); data reduction: *SAINT*; program(s) used to solve structure: *SHELXS97* (Sheldrick, 2008 ▶); program(s) used to refine structure: *SHELXL97* (Sheldrick, 2008 ▶); molecular graphics: *SHELXTL* (Sheldrick, 2008 ▶); software used to prepare material for publication: *SHELXL97*.

## Supplementary Material

Crystal structure: contains datablocks I, global. DOI: 10.1107/S1600536810047203/pv2354sup1.cif

Structure factors: contains datablocks I. DOI: 10.1107/S1600536810047203/pv2354Isup2.hkl

Additional supplementary materials:  crystallographic information; 3D view; checkCIF report

## Figures and Tables

**Table 1:** Hydrogen-bond geometry (Å, °)

*D*—H⋯*A*	*D*—H	H⋯*A*	*D*⋯*A*	*D*—H⋯*A*
N1—H1⋯O8^i^	0.86 (5)	1.95 (5)	2.806 (6)	176 (7)
O1*W*—H1*W*⋯O8^ii^	0.82 (5)	2.08 (5)	2.885 (6)	166 (5)
N4—H2⋯O3^iii^	0.86 (4)	1.93 (3)	2.785 (5)	172 (7)
O1*W*—H2*W*⋯O3^ii^	0.82 (5)	2.01 (4)	2.822 (5)	170 (4)
O1*W*—H2*W*⋯O4^ii^	0.82 (5)	2.58 (5)	3.048 (5)	118 (5)
O2*W*—H3*W*⋯O16^iv^	0.81 (4)	1.92 (5)	2.730 (5)	176 (8)
O2*W*—H4*W*⋯O3^ii^	0.80 (4)	2.49 (5)	2.897 (5)	113 (4)
O3*W*—H5*W*⋯O5*W*	0.82 (7)	1.89 (7)	2.692 (7)	169 (7)
O3*W*—H6*W*⋯O6^v^	0.81 (3)	2.57 (4)	3.336 (6)	158 (8)
O4*W*—H7*W*⋯O5*W*	0.82 (4)	1.98 (4)	2.752 (6)	158 (6)
O4*W*—H8*W*⋯O2*W*	0.81 (5)	2.57 (7)	3.167 (6)	132 (6)
O5*W*—H9*W*⋯O8^vi^	0.85 (5)	1.90 (5)	2.737 (6)	169 (5)
O5*W*—H10*W*⋯O16^iv^	0.86 (5)	1.97 (6)	2.787 (6)	159 (7)
C3—H3⋯O6^iii^	0.93	2.46	3.224 (7)	140

Symmetry codes: (i) 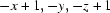; (ii) 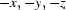; (iii) 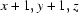; (iv) 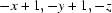; (v) 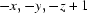; (vi) 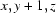.

**Fig. 1.**
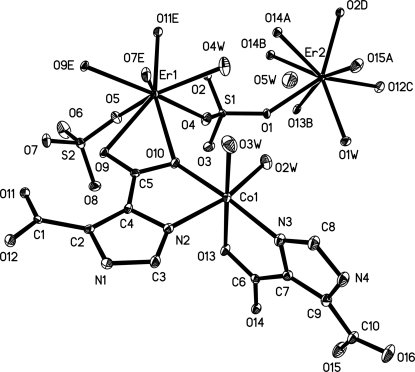
The molecular structure showing the atomic-numbering scheme and displacement ellipsoids drawn at the 30% probability level. Symmetry codes: (A) -1 + x, y, z; (B) -x, -y, -z; (C) -1 + x, y, -1 + z; (D) -1 - x, -y, -z; (E) -x, -y, 1 - z.

**Fig. 2.**
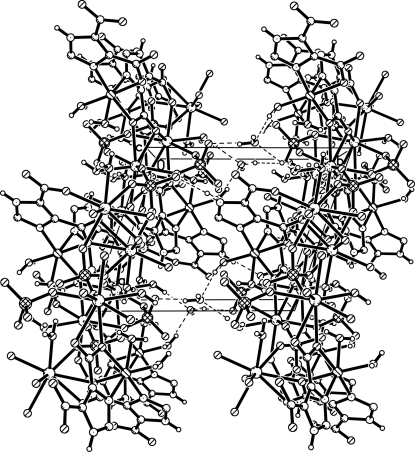
A view of the three-dimensional structure of the title compound, the hydrogen bonding interactions showed as broken lines.

## supplementary crystallographic information

### Comment

In the past few years, there has been a tremendous increase of interset in lanthanide-transition metal heterometallic complexs with bridging multifunctionnal organic ligands because of their impressive topological structures as well aso due to their versatile applications in ion exchange, magnetism, bimetallic catalysis and luminescent probes (Cheng *et al.*, 2006; Kuang *et al.*, 2007; Sun *et al.*, 2006; Zhu *et al.*, 2010). As an extension of this research, the structure of the title compound, a new heterometallic coordination polymer, (I), has been determined which is presented in this artcle.

The asymmetric unite of the title compound (Fig. 1), contains a Co^II^ ion, two Er^III^ ions, two imidazole-4,5-dicarboxylate (imdc) ligands, two SO_4_^2-^ anions, four coordinated water molecules and one lattice water molecule. The Co^II^ ion is six-coordinated with two O atoms from two coordinated water molecules, two O atoms and two N atoms from two imdc ligands, to give a slightly distorted octahedral geometry. Both Er^III^ ions are eight-coordinated in a bicapped trigonal prismatic coordination geometry. One Er^III^ ion is coordinated by four O atoms from two idmc ligands, three O atoms from three SO_4_^2-^ anions and one water molecule; the other Er^III^ ion is bonded to five O atoms from three imidazole-4, 5-dicarboxylate ligands, two O atoms from two SO_4_^2-^ anions as well as one coordinated water molecule. These metal coordination units are connected by bridging imdc ligands and sulfate anions, generating a two-dimensional heterometallic layer. The two-dimensional layers are stacked along *b* axis *via* hydrogen-bonding interactions between water molecules, SO_4_^2-^ anions, and imdc ligands to generate the three-dimensional framework (Tab. 1 and Fig. 2).

### Experimental

A mixture of CoSO_4_.7H_2_O (0.141 g, 0.5 mmol), Er_2_O_3_ (0.098 g, 0.25 mmol), imidazole-4,5-dicarboxylic acid (0.156 g, 1 mmol), and H_2_O (7 ml) was sealed in a 20 ml Teflon-lined reaction vessel at 443 K for 5 days then slowly cooled to room temperature. The product was collected by filtration, washed with water and air-dried. Red block crystals suitable for X-ray analysis were obtained.

### Refinement

H atoms bonded to C atoms were positioned geometrically and refined as riding, with C—H = 0.93 Å and *U*_iso_(H) = 1.2 *U*_eq_(C). H atoms bonded to N atoms and H atoms of water molecules were found from difference Fourier maps and refined isotropically with restraints: N—H = 0.87 Å, O—H = 0.82 or 0.86 Å and *U*_iso_(H) = 1.5 *U*_eq_(N, O). In the final difference map, the largest residual electron density and the deepest hole are located at 0.96 and 0.85 Å, respectively from Er2.

### Crystal data

**Table:**

[CoEr_2_(C_5_H_2_N_2_O_4_)_2_(SO_4_)_2_(H_2_O)_4_]·H_2_O	*Z* = 2
*M**_r_* = 983.84	*F*(000) = 930
Triclinic, *P*1	*D*_x_ = 2.799 Mg m^−^^3^
Hall symbol: -P 1	Mo *K*α radiation, λ = 0.71073 Å
*a* = 9.0512 (5) Å	Cell parameters from 4400 reflections
*b* = 10.6827 (6) Å	θ = 2.4–28.0°
*c* = 12.8945 (8) Å	µ = 8.12 mm^−^^1^
α = 92.955 (1)°	*T* = 296 K
β = 97.054 (1)°	Block, red
γ = 108.612 (1)°	0.20 × 0.18 × 0.15 mm
*V* = 1167.17 (12) Å^3^	

### Data collection

**Table:**

Bruker APEXII area-detector diffractometer	4123 independent reflections
Radiation source: fine-focus sealed tube	3820 reflections with *I* > 2σ(*I*)
graphite	*R*_int_ = 0.017
φ and ω scan	θ_max_ = 25.2°, θ_min_ = 1.6°
Absorption correction: multi-scan (*SADABS*; Sheldrick, 1996)	*h* = −10→5
*T*_min_ = 0.215, *T*_max_ = 0.296	*k* = −12→12
6026 measured reflections	*l* = −15→15

### Refinement

**Table:**

Refinement on *F*^2^	Primary atom site location: structure-invariant direct methods
Least-squares matrix: full	Secondary atom site location: difference Fourier map
*R*[*F*^2^ > 2σ(*F*^2^)] = 0.023	Hydrogen site location: inferred from neighbouring sites
*wR*(*F*^2^) = 0.055	H atoms treated by a mixture of independent and constrained refinement
*S* = 1.03	*w* = 1/[σ^2^(*F*_o_^2^) + (0.0283*P*)^2^ + 1.940*P*] where *P* = (*F*_o_^2^ + 2*F*_c_^2^)/3
4123 reflections	(Δ/σ)_max_ = 0.001
397 parameters	Δρ_max_ = 0.87 e Å^−^^3^
12 restraints	Δρ_min_ = −1.35 e Å^−^^3^

### Special details

**Table:**

Geometry. All e.s.d.'s (except the e.s.d. in the dihedral angle between two l.s. planes) are estimated using the full covariance matrix. The cell e.s.d.'s are taken into account individually in the estimation of e.s.d.'s in distances, angles and torsion angles; correlations between e.s.d.'s in cell parameters are only used when they are defined by crystal symmetry. An approximate (isotropic) treatment of cell e.s.d.'s is used for estimating e.s.d.'s involving l.s. planes.
Refinement. Refinement of *F*^2^ against ALL reflections. The weighted *R*-factor *wR* and goodness of fit *S* are based on *F*^2^, conventional *R*-factors *R* are based on *F*, with *F* set to zero for negative *F*^2^. The threshold expression of *F*^2^ > σ(*F*^2^) is used only for calculating *R*-factors(gt) *etc*. and is not relevant to the choice of reflections for refinement. *R*-factors based on *F*^2^ are statistically about twice as large as those based on *F*, and *R*- factors based on ALL data will be even larger.

### Fractional atomic coordinates and isotropic or equivalent isotropic displacement parameters (Å^2^)

**Table:**

	*x*	*y*	*z*	*U*_iso_*/*U*_eq_	
Er1	−0.09070 (2)	0.049642 (19)	0.366467 (14)	0.01288 (7)	
Er2	−0.37193 (2)	0.088827 (18)	−0.105666 (14)	0.01193 (7)	
Co1	0.34879 (7)	0.28628 (6)	0.27282 (4)	0.01585 (14)	
S1	−0.22681 (13)	−0.07707 (11)	0.09734 (8)	0.0129 (2)	
S2	−0.07687 (14)	−0.27513 (11)	0.40191 (9)	0.0170 (2)	
C1	0.4802 (5)	0.0827 (4)	0.6454 (3)	0.0145 (9)	
C2	0.5054 (5)	0.1537 (4)	0.5507 (3)	0.0152 (9)	
C3	0.6350 (6)	0.2989 (5)	0.4510 (4)	0.0212 (10)	
H3	0.7175	0.3621	0.4271	0.025*	
C4	0.4062 (5)	0.1634 (4)	0.4630 (3)	0.0150 (9)	
C5	0.2387 (5)	0.1004 (4)	0.4232 (3)	0.0144 (9)	
C6	0.5165 (5)	0.1950 (4)	0.1209 (3)	0.0144 (9)	
C7	0.5691 (6)	0.3402 (5)	0.1209 (4)	0.0177 (10)	
C8	0.5776 (6)	0.5343 (5)	0.1817 (4)	0.0235 (11)	
H8	0.5609	0.6028	0.2209	0.028*	
C9	0.6679 (6)	0.4298 (4)	0.0658 (4)	0.0196 (10)	
C10	0.7520 (6)	0.4192 (5)	−0.0250 (4)	0.0218 (11)	
N1	0.6495 (5)	0.2411 (4)	0.5397 (3)	0.0190 (9)	
N2	0.4904 (5)	0.2554 (4)	0.4025 (3)	0.0169 (8)	
N3	0.5133 (5)	0.4078 (4)	0.1927 (3)	0.0189 (9)	
N4	0.6703 (5)	0.5521 (4)	0.1067 (3)	0.0249 (9)	
O1	−0.2200 (4)	0.0083 (3)	0.0104 (2)	0.0208 (7)	
O2	−0.3866 (4)	−0.1266 (3)	0.1256 (3)	0.0224 (7)	
O3	−0.1750 (4)	−0.1884 (3)	0.0667 (3)	0.0245 (8)	
O4	−0.1162 (4)	0.0023 (3)	0.1883 (2)	0.0229 (8)	
O5	−0.1393 (4)	−0.1746 (3)	0.3560 (2)	0.0218 (7)	
O6	−0.1995 (5)	−0.4018 (4)	0.3964 (3)	0.0407 (10)	
O7	−0.0089 (4)	−0.2307 (3)	0.5147 (2)	0.0235 (8)	
O8	0.0526 (4)	−0.2828 (4)	0.3446 (3)	0.0278 (8)	
O9	0.1450 (4)	0.0157 (3)	0.4693 (2)	0.0171 (7)	
O10	0.1848 (4)	0.1367 (3)	0.3383 (2)	0.0182 (7)	
O11	0.3504 (4)	−0.0057 (3)	0.6478 (2)	0.0183 (7)	
O12	0.5935 (4)	0.1177 (3)	0.7192 (2)	0.0235 (8)	
O13	0.4190 (4)	0.1442 (3)	0.1823 (2)	0.0178 (7)	
O14	0.5640 (4)	0.1192 (3)	0.0648 (2)	0.0164 (7)	
O15	0.7324 (5)	0.3048 (3)	−0.0682 (3)	0.0322 (9)	
O16	0.8340 (5)	0.5232 (3)	−0.0549 (3)	0.0367 (10)	
H2	0.726 (7)	0.631 (3)	0.097 (5)	0.055*	
H1	0.739 (4)	0.250 (6)	0.576 (5)	0.055*	
O1W	−0.1359 (4)	0.1275 (4)	−0.1711 (3)	0.0261 (8)	
H1W	−0.120 (7)	0.159 (6)	−0.227 (3)	0.039*	
H2W	−0.050 (4)	0.140 (6)	−0.135 (4)	0.039*	
O2W	0.1566 (5)	0.2533 (4)	0.1512 (3)	0.0285 (8)	
H3W	0.164 (8)	0.320 (4)	0.122 (4)	0.043*	
H4W	0.121 (7)	0.182 (3)	0.118 (4)	0.043*	
O3W	0.2893 (6)	0.4288 (4)	0.3606 (3)	0.0376 (10)	
H5W	0.226 (7)	0.456 (7)	0.326 (5)	0.056*	
H6W	0.290 (9)	0.416 (7)	0.422 (2)	0.056*	
O4W	−0.1271 (5)	0.2319 (4)	0.2783 (3)	0.0366 (10)	
H7W	−0.072 (7)	0.310 (3)	0.286 (5)	0.055*	
H8W	−0.106 (8)	0.223 (7)	0.220 (3)	0.055*	
O5W	0.0527 (6)	0.4842 (4)	0.2458 (3)	0.0433 (11)	
H9W	0.044 (9)	0.557 (4)	0.269 (5)	0.065*	
H10W	0.079 (9)	0.499 (7)	0.185 (3)	0.065*	

### Atomic displacement parameters (Å^2^)

**Table:**

	*U*^11^	*U*^22^	*U*^33^	*U*^12^	*U*^13^	*U*^23^
Er1	0.01014 (12)	0.01703 (11)	0.01108 (11)	0.00352 (8)	0.00105 (8)	0.00507 (8)
Er2	0.01019 (12)	0.01510 (11)	0.01112 (11)	0.00425 (8)	0.00202 (8)	0.00503 (8)
Co1	0.0167 (3)	0.0187 (3)	0.0132 (3)	0.0060 (3)	0.0037 (2)	0.0058 (2)
S1	0.0105 (6)	0.0177 (5)	0.0113 (5)	0.0056 (4)	0.0012 (4)	0.0046 (4)
S2	0.0165 (6)	0.0160 (5)	0.0170 (5)	0.0042 (5)	−0.0009 (5)	0.0020 (4)
C1	0.014 (2)	0.024 (2)	0.010 (2)	0.013 (2)	0.0026 (18)	0.0040 (18)
C2	0.009 (2)	0.021 (2)	0.015 (2)	0.0033 (19)	0.0025 (18)	0.0037 (18)
C3	0.017 (3)	0.025 (3)	0.021 (2)	0.003 (2)	0.005 (2)	0.010 (2)
C4	0.017 (3)	0.021 (2)	0.009 (2)	0.007 (2)	0.0039 (18)	0.0029 (17)
C5	0.013 (2)	0.021 (2)	0.012 (2)	0.0077 (19)	0.0023 (18)	0.0007 (18)
C6	0.010 (2)	0.018 (2)	0.013 (2)	0.0030 (19)	−0.0023 (18)	0.0039 (18)
C7	0.015 (2)	0.019 (2)	0.018 (2)	0.0043 (19)	0.0024 (19)	0.0044 (18)
C8	0.031 (3)	0.018 (2)	0.023 (2)	0.007 (2)	0.011 (2)	0.0044 (19)
C9	0.020 (3)	0.016 (2)	0.021 (2)	0.003 (2)	0.004 (2)	0.0038 (19)
C10	0.022 (3)	0.019 (2)	0.023 (2)	0.003 (2)	0.009 (2)	0.006 (2)
N1	0.008 (2)	0.026 (2)	0.0180 (19)	0.0005 (17)	−0.0027 (16)	0.0045 (17)
N2	0.014 (2)	0.021 (2)	0.0164 (19)	0.0049 (16)	0.0034 (16)	0.0049 (16)
N3	0.023 (2)	0.016 (2)	0.0168 (19)	0.0049 (17)	0.0050 (17)	0.0012 (15)
N4	0.030 (3)	0.016 (2)	0.027 (2)	0.0035 (19)	0.0112 (19)	0.0055 (18)
O1	0.0156 (18)	0.0305 (19)	0.0197 (16)	0.0097 (15)	0.0039 (14)	0.0155 (14)
O2	0.0127 (18)	0.0340 (19)	0.0241 (17)	0.0097 (15)	0.0060 (14)	0.0164 (15)
O3	0.028 (2)	0.0163 (17)	0.0333 (19)	0.0085 (15)	0.0146 (16)	0.0047 (14)
O4	0.0192 (19)	0.0291 (19)	0.0143 (16)	0.0004 (15)	−0.0006 (14)	0.0032 (14)
O5	0.0226 (19)	0.0201 (17)	0.0219 (17)	0.0079 (15)	−0.0028 (14)	0.0022 (14)
O6	0.030 (2)	0.023 (2)	0.054 (3)	−0.0059 (17)	−0.0115 (19)	0.0110 (18)
O7	0.034 (2)	0.0225 (18)	0.0148 (16)	0.0116 (16)	0.0009 (15)	0.0048 (13)
O8	0.026 (2)	0.044 (2)	0.0189 (17)	0.0186 (17)	0.0052 (15)	0.0015 (15)
O9	0.0104 (16)	0.0256 (18)	0.0136 (15)	0.0025 (14)	0.0031 (13)	0.0080 (13)
O10	0.0124 (17)	0.0270 (18)	0.0146 (15)	0.0044 (14)	0.0032 (13)	0.0082 (13)
O11	0.0102 (17)	0.0265 (18)	0.0170 (16)	0.0035 (14)	0.0018 (13)	0.0079 (13)
O12	0.0103 (17)	0.038 (2)	0.0179 (16)	0.0024 (15)	−0.0020 (14)	0.0098 (15)
O13	0.0208 (18)	0.0150 (16)	0.0169 (15)	0.0024 (14)	0.0082 (14)	0.0045 (13)
O14	0.0190 (18)	0.0136 (15)	0.0161 (15)	0.0036 (13)	0.0051 (13)	0.0014 (12)
O15	0.041 (2)	0.0173 (18)	0.036 (2)	0.0022 (16)	0.0194 (18)	0.0018 (15)
O16	0.050 (3)	0.0216 (19)	0.040 (2)	0.0056 (18)	0.026 (2)	0.0105 (16)
O1W	0.0148 (19)	0.046 (2)	0.0222 (18)	0.0137 (17)	0.0050 (15)	0.0139 (17)
O2W	0.032 (2)	0.030 (2)	0.0228 (19)	0.0108 (19)	−0.0028 (16)	0.0054 (16)
O3W	0.052 (3)	0.035 (2)	0.031 (2)	0.024 (2)	0.001 (2)	0.0002 (18)
O4W	0.047 (3)	0.025 (2)	0.033 (2)	0.0063 (19)	−0.001 (2)	0.0113 (18)
O5W	0.063 (3)	0.035 (2)	0.043 (2)	0.025 (2)	0.025 (2)	0.009 (2)

### Geometric parameters (Å, °)

**Table:**

Er1—O11^i^	2.227 (3)	C3—H3	0.9300
Er1—O7^i^	2.268 (3)	C4—N2	1.380 (6)
Er1—O5	2.286 (3)	C4—C5	1.462 (6)
Er1—O4	2.294 (3)	C5—O9	1.258 (5)
Er1—O9^i^	2.324 (3)	C5—O10	1.272 (5)
Er1—O4W	2.396 (4)	C6—O14	1.267 (5)
Er1—O10	2.447 (3)	C6—O13	1.269 (5)
Er1—O9	2.508 (3)	C6—C7	1.470 (6)
Er1—C5	2.848 (4)	C6—Er2^v^	2.879 (4)
Er2—O15^ii^	2.198 (3)	C7—C9	1.378 (7)
Er2—O12^iii^	2.291 (3)	C7—N3	1.383 (6)
Er2—O1	2.292 (3)	C8—N3	1.313 (6)
Er2—O1W	2.316 (4)	C8—N4	1.338 (6)
Er2—O2^iv^	2.333 (3)	C8—H8	0.9300
Er2—O14^ii^	2.373 (3)	C9—N4	1.376 (6)
Er2—O14^v^	2.474 (3)	C9—C10	1.492 (7)
Er2—O13^v^	2.514 (3)	C10—O16	1.234 (6)
Er2—C6^v^	2.879 (4)	C10—O15	1.266 (6)
Er2—Er2^iv^	3.9596 (4)	N1—H1	0.86 (5)
Co1—N3	2.062 (4)	N4—H2	0.86 (4)
Co1—N2	2.085 (4)	O2—Er2^iv^	2.333 (3)
Co1—O3W	2.093 (4)	O7—Er1^i^	2.268 (3)
Co1—O10	2.099 (3)	O9—Er1^i^	2.324 (3)
Co1—O2W	2.120 (4)	O11—Er1^i^	2.227 (3)
Co1—O13	2.165 (3)	O12—Er2^vi^	2.291 (3)
S1—O3	1.464 (3)	O13—Er2^v^	2.514 (3)
S1—O4	1.471 (3)	O14—Er2^vii^	2.373 (3)
S1—O2	1.471 (3)	O14—Er2^v^	2.474 (3)
S1—O1	1.477 (3)	O15—Er2^vii^	2.198 (3)
S2—O6	1.443 (4)	O1W—H1W	0.82 (5)
S2—O5	1.481 (3)	O1W—H2W	0.82 (5)
S2—O8	1.482 (4)	O2W—H3W	0.81 (4)
S2—O7	1.497 (3)	O2W—H4W	0.80 (4)
C1—O11	1.255 (6)	O3W—H5W	0.82 (7)
C1—O12	1.256 (5)	O3W—H6W	0.81 (3)
C1—C2	1.474 (6)	O4W—H7W	0.82 (4)
C2—N1	1.369 (6)	O4W—H8W	0.81 (5)
C2—C4	1.385 (6)	O5W—H9W	0.85 (5)
C3—N2	1.304 (6)	O5W—H10W	0.86 (5)
C3—N1	1.339 (6)		
			
O11^i^—Er1—O7^i^	103.78 (12)	O2W—Co1—O13	87.26 (13)
O11^i^—Er1—O5	87.24 (12)	O3—S1—O4	108.4 (2)
O7^i^—Er1—O5	140.03 (11)	O3—S1—O2	110.1 (2)
O11^i^—Er1—O4	89.33 (12)	O4—S1—O2	109.7 (2)
O7^i^—Er1—O4	137.77 (12)	O3—S1—O1	109.2 (2)
O5—Er1—O4	79.46 (12)	O4—S1—O1	107.83 (19)
O11^i^—Er1—O9^i^	77.27 (11)	O2—S1—O1	111.55 (19)
O7^i^—Er1—O9^i^	71.65 (11)	O6—S2—O5	111.1 (2)
O5—Er1—O9^i^	73.70 (11)	O6—S2—O8	112.1 (2)
O4—Er1—O9^i^	150.39 (11)	O5—S2—O8	107.4 (2)
O11^i^—Er1—O4W	77.71 (14)	O6—S2—O7	108.6 (2)
O7^i^—Er1—O4W	73.79 (12)	O5—S2—O7	109.60 (18)
O5—Er1—O4W	145.94 (13)	O8—S2—O7	107.9 (2)
O4—Er1—O4W	70.10 (13)	O11—C1—O12	124.8 (4)
O9^i^—Er1—O4W	130.42 (13)	O11—C1—C2	119.4 (4)
O11^i^—Er1—O10	162.82 (11)	O12—C1—C2	115.8 (4)
O7^i^—Er1—O10	77.34 (12)	N1—C2—C4	104.3 (4)
O5—Er1—O10	102.96 (12)	N1—C2—C1	121.7 (4)
O4—Er1—O10	79.20 (11)	C4—C2—C1	133.7 (4)
O9^i^—Er1—O10	118.64 (10)	N2—C3—N1	111.4 (4)
O4W—Er1—O10	86.33 (13)	N2—C3—H3	124.3
O11^i^—Er1—O9	145.10 (10)	N1—C3—H3	124.3
O7^i^—Er1—O9	75.86 (11)	N2—C4—C2	109.5 (4)
O5—Er1—O9	73.80 (11)	N2—C4—C5	115.7 (4)
O4—Er1—O9	114.74 (11)	C2—C4—C5	134.8 (4)
O9^i^—Er1—O9	69.47 (12)	O9—C5—O10	118.6 (4)
O4W—Er1—O9	132.82 (13)	O9—C5—C4	123.8 (4)
O10—Er1—O9	52.05 (10)	O10—C5—C4	117.6 (4)
O11^i^—Er1—C5	169.83 (11)	O9—C5—Er1	61.7 (2)
O7^i^—Er1—C5	71.00 (13)	O10—C5—Er1	58.9 (2)
O5—Er1—C5	91.51 (12)	C4—C5—Er1	163.6 (3)
O4—Er1—C5	100.37 (12)	O14—C6—O13	118.8 (4)
O9^i^—Er1—C5	92.68 (11)	O14—C6—C7	124.0 (4)
O4W—Er1—C5	108.36 (14)	O13—C6—C7	117.2 (4)
O10—Er1—C5	26.44 (11)	O14—C6—Er2^v^	58.8 (2)
O9—Er1—C5	26.19 (11)	O13—C6—Er2^v^	60.7 (2)
O15^ii^—Er2—O12^iii^	89.96 (13)	C7—C6—Er2^v^	172.0 (3)
O15^ii^—Er2—O1	102.94 (13)	C9—C7—N3	109.4 (4)
O12^iii^—Er2—O1	139.27 (12)	C9—C7—C6	134.6 (4)
O15^ii^—Er2—O1W	79.51 (14)	N3—C7—C6	116.0 (4)
O12^iii^—Er2—O1W	69.98 (12)	N3—C8—N4	111.3 (4)
O1—Er2—O1W	74.63 (12)	N3—C8—H8	124.3
O15^ii^—Er2—O2^iv^	85.40 (13)	N4—C8—H8	124.3
O12^iii^—Er2—O2^iv^	78.38 (12)	N4—C9—C7	104.8 (4)
O1—Er2—O2^iv^	140.26 (11)	N4—C9—C10	120.1 (4)
O1W—Er2—O2^iv^	144.73 (11)	C7—C9—C10	134.9 (4)
O15^ii^—Er2—O14^ii^	77.81 (12)	O16—C10—O15	124.0 (5)
O12^iii^—Er2—O14^ii^	149.10 (12)	O16—C10—C9	117.7 (4)
O1—Er2—O14^ii^	71.57 (11)	O15—C10—C9	118.3 (4)
O1W—Er2—O14^ii^	133.35 (12)	C3—N1—C2	108.8 (4)
O2^iv^—Er2—O14^ii^	72.50 (11)	C3—N1—H1	123 (5)
O15^ii^—Er2—O14^v^	146.41 (12)	C2—N1—H1	127 (5)
O12^iii^—Er2—O14^v^	111.88 (11)	C3—N2—C4	106.0 (4)
O1—Er2—O14^v^	77.45 (11)	C3—N2—Co1	141.3 (3)
O1W—Er2—O14^v^	131.03 (12)	C4—N2—Co1	112.6 (3)
O2^iv^—Er2—O14^v^	75.04 (11)	C8—N3—C7	106.0 (4)
O14^ii^—Er2—O14^v^	70.43 (11)	C8—N3—Co1	139.7 (3)
O15^ii^—Er2—O13^v^	161.49 (12)	C7—N3—Co1	114.1 (3)
O12^iii^—Er2—O13^v^	80.41 (11)	C8—N4—C9	108.5 (4)
O1—Er2—O13^v^	75.46 (11)	C8—N4—H2	120 (4)
O1W—Er2—O13^v^	82.34 (12)	C9—N4—H2	131 (4)
O2^iv^—Er2—O13^v^	107.80 (11)	S1—O1—Er2	143.0 (2)
O14^ii^—Er2—O13^v^	118.04 (10)	S1—O2—Er2^iv^	142.13 (19)
O14^v^—Er2—O13^v^	51.89 (10)	S1—O4—Er1	142.7 (2)
O15^ii^—Er2—C6^v^	171.02 (13)	S2—O5—Er1	141.0 (2)
O12^iii^—Er2—C6^v^	98.50 (12)	S2—O7—Er1^i^	143.41 (19)
O1—Er2—C6^v^	72.49 (12)	C5—O9—Er1^i^	143.6 (3)
O1W—Er2—C6^v^	106.14 (13)	C5—O9—Er1	92.2 (3)
O2^iv^—Er2—C6^v^	93.33 (12)	Er1^i^—O9—Er1	110.53 (12)
O14^ii^—Er2—C6^v^	93.33 (11)	C5—O10—Co1	115.3 (3)
O14^v^—Er2—C6^v^	26.00 (11)	C5—O10—Er1	94.6 (3)
O13^v^—Er2—C6^v^	26.10 (11)	Co1—O10—Er1	144.42 (15)
O15^ii^—Er2—Er2^iv^	113.19 (9)	C1—O11—Er1^i^	144.9 (3)
O12^iii^—Er2—Er2^iv^	138.32 (8)	C1—O12—Er2^vi^	136.0 (3)
O1—Er2—Er2^iv^	71.02 (8)	C6—O13—Co1	114.4 (3)
O1W—Er2—Er2^iv^	145.23 (8)	C6—O13—Er2^v^	93.2 (3)
O2^iv^—Er2—Er2^iv^	70.03 (8)	Co1—O13—Er2^v^	150.77 (15)
O14^ii^—Er2—Er2^iv^	36.06 (7)	C6—O14—Er2^vii^	141.8 (3)
O14^v^—Er2—Er2^iv^	34.37 (7)	C6—O14—Er2^v^	95.2 (3)
O13^v^—Er2—Er2^iv^	84.04 (7)	Er2^vii^—O14—Er2^v^	109.57 (11)
C6^v^—Er2—Er2^iv^	58.22 (9)	C10—O15—Er2^vii^	158.3 (3)
N3—Co1—N2	102.00 (15)	Er2—O1W—H1W	123 (4)
N3—Co1—O3W	100.19 (16)	Er2—O1W—H2W	125 (4)
N2—Co1—O3W	92.08 (15)	H1W—O1W—H2W	108 (6)
N3—Co1—O10	170.48 (14)	Co1—O2W—H3W	111 (5)
N2—Co1—O10	78.70 (13)	Co1—O2W—H4W	119 (5)
O3W—Co1—O10	89.25 (16)	H3W—O2W—H4W	119 (6)
N3—Co1—O2W	95.07 (15)	Co1—O3W—H5W	112 (5)
N2—Co1—O2W	160.56 (15)	Co1—O3W—H6W	113 (5)
O3W—Co1—O2W	94.00 (16)	H5W—O3W—H6W	126 (7)
O10—Co1—O2W	82.93 (14)	Er1—O4W—H7W	130 (5)
N3—Co1—O13	77.96 (13)	Er1—O4W—H8W	107 (5)
N2—Co1—O13	87.28 (14)	H7W—O4W—H8W	91 (7)
O3W—Co1—O13	177.87 (16)	H9W—O5W—H10W	104 (7)
O10—Co1—O13	92.62 (12)		

Symmetry codes: (i) −*x*, −*y*, −*z*+1; (ii) *x*−1, *y*, *z*; (iii) *x*−1, *y*, *z*−1; (iv) −*x*−1, −*y*, −*z*; (v) −*x*, −*y*, −*z*; (vi) *x*+1, *y*, *z*+1; (vii) *x*+1, *y*, *z*.

### Hydrogen-bond geometry (Å, °)

**Table:**

*D*—H···*A*	*D*—H	H···*A*	*D*···*A*	*D*—H···*A*
N1—H1···O8^viii^	0.86 (5)	1.95 (5)	2.806 (6)	176 (7)
O1W—H1W···O8^ix^	0.82 (5)	2.08 (5)	2.885 (6)	166 (5)
N4—H2···O3^x^	0.86 (4)	1.93 (3)	2.785 (5)	172 (7)
O1W—H2W···O3^ix^	0.82 (5)	2.01 (4)	2.822 (5)	170 (4)
O1W—H2W···O4^ix^	0.82 (5)	2.58 (5)	3.048 (5)	118 (5)
O2W—H3W···O16^xi^	0.81 (4)	1.92 (5)	2.730 (5)	176 (8)
O2W—H4W···O3^ix^	0.80 (4)	2.49 (5)	2.897 (5)	113 (4)
O3W—H5W···O5W	0.82 (7)	1.89 (7)	2.692 (7)	169 (7)
O3W—H6W···O6^i^	0.81 (3)	2.57 (4)	3.336 (6)	158 (8)
O4W—H7W···O5W	0.82 (4)	1.98 (4)	2.752 (6)	158 (6)
O4W—H8W···O2W	0.81 (5)	2.57 (7)	3.167 (6)	132 (6)
O5W—H9W···O8^xii^	0.85 (5)	1.90 (5)	2.737 (6)	169 (5)
O5W—H10W···O16^xi^	0.86 (5)	1.97 (6)	2.787 (6)	159 (7)
C3—H3···O6^x^	0.93	2.46	3.224 (7)	140

Symmetry codes: (viii) −*x*+1, −*y*, −*z*+1; (ix) −*x*, −*y*, −*z*; (x) *x*+1, *y*+1, *z*; (xi) −*x*+1, −*y*+1, −*z*; (i) −*x*, −*y*, −*z*+1; (xii) *x*, *y*+1, *z*.
